# Orbital rhabdomyosarcoma in an adult: a misleading presentation

**DOI:** 10.1016/j.radcr.2026.01.101

**Published:** 2026-02-28

**Authors:** Mohamed Bouallou, Issam Berrajaa, Achraf Amine Sbai, Mohammed El Magroud, Drissia Benfadil, Amal Bennani, Azeddine Lachkar

**Affiliations:** a Faculty of Medicine and Pharmacy, Mohammed First University, Oujda, Morocco; b Department of Otorhinolaryngology, Mohammed VI University Hospital, Oujda, Morocco; c Laboratory of Oto-Neuro-Ophthalmology (LORNO), Faculty of Medicine and Pharmacy, Mohammed First University, Oujda, Morocco; d Mohammed First University, Faculty of Medicine and Pharmacy, LAMCESM, Oujda, Morocco; e Department of Pathology, Mohammed VI University Hospital, Mohammed First University, Oujda, Morocco

**Keywords:** Rhabdomyosarcoma, Orbit, Adult, Hyposmia, Nasal obstruction, MRI, Case report

## Abstract

Rhabdomyosarcoma is the most common malignant mesenchymal tumor in children, whereas its occurrence in adults is exceptionally rare. When it occurs in this population, initial presentation with nonspecific rhinologic symptoms is unusual and may delay recognition of the underlying malignancy. We report the case of a 29-year-old woman who presented with progressive right-sided nasal obstruction and hyposmia, subsequently complicated by the rapid onset of unilateral proptosis. Cross-sectional imaging revealed a poorly circumscribed ethmoido-orbito-nasal mass with skull base erosion and orbital invasion, exhibiting aggressive radiologic features that initially raised suspicion for esthesioneuroblastoma. Histopathological examination ultimately confirmed alveolar rhabdomyosarcoma, staged as T2BN1M0 (IRS Group III). The patient underwent multimodal treatment combining VAC chemotherapy and conformal radiotherapy (45 Gy), resulting in an excellent clinical and radiologic response, with complete resolution of proptosis and significant visual recovery at 1-year follow-up. This case emphasizes the diagnostic challenges of adult orbital rhabdomyosarcoma with sinonasal extension and highlights the pivotal role of advanced imaging and histopathology in guiding accurate diagnosis and appropriate management. The rarity and unusual clinical presentation of this case underscore its value for publication.

## Introduction

Rhabdomyosarcoma predominantly affects the pediatric population, whereas its diagnosis in adults remains exceedingly uncommon [1]. In this population, the diagnosis is often delayed, as the disease may present with atypical or nonspecific symptoms that can mimic benign or inflammatory conditions, leading to advanced-stage detection.

Orbital involvement is uncommon in adults and classically manifests as rapidly progressive unilateral proptosis. In contrast, initial presentation dominated by nonspecific sinonasal symptoms, such as nasal obstruction or hyposmia, is distinctly unusual in adult orbital RMS and remains underreported in the literature [2].

Definitive diagnosis requires histopathological confirmation, supported by immunohistochemical positivity for muscle-specific markers such as Desmin and Myogenin. Four histopathological subtypes of rhabdomyosarcoma have been described: pleomorphic RMS, alveolar RMS, botryoid RMS, and embryonal RMS [3]. Among these, the alveolar subtype, most frequently identified in adolescents and young adults, is associated with an unfavorable prognosis, partly due to the PAX3–FOXO1 fusion gene, which drives a more aggressive clinical behavior [4].

Cross-sectional imaging plays a pivotal role in raising early suspicion for malignancy in these atypical cases. Magnetic resonance imaging constitutes the cornerstone of radiologic evaluation, providing superior soft-tissue contrast and detailed assessment of local extension, while computed tomography offers complementary information on osseous involvement. Optimal management of RMS relies on a multidisciplinary, multimodal approach integrating systemic chemotherapy, surgery, and/or radiotherapy to achieve local control and improve survival outcomes [5].

There is a paucity of published reports focusing specifically on adult orbital rhabdomyosarcoma presenting with misleading symptoms, particularly those emphasizing radiologic diagnostic pitfalls and differential considerations.

We therefore report a rare case of orbital alveolar rhabdomyosarcoma in an adult woman who initially presented with isolated sinonasal symptoms, highlighting the diagnostic challenges, key imaging features, and the critical role of radiologic–pathologic correlation in guiding timely and appropriate management.

## Case presentation

A 29-year-old woman with no significant medical history was referred to our Otorhinolaryngology Department for evaluation of progressive right-sided nasal obstruction associated with hyposmia evolving over 7 weeks. She denied epistaxis, diplopia, facial pain, or visual loss. There was no history of malignancy, radiation exposure, or familial cancer predisposition. However, she reported chronic occupational exposure to wood dust.

Initial management with topical corticosteroids and saline irrigations prescribed for presumed chronic rhinosinusitis, failed to improve symptoms. Two weeks before referral, the abrupt onset of rapidly progressive right-sided proptosis associated with mild periorbital edema, prompting evaluation to our tertiary care center (Table 1).

**Table 1:** Timeline of clinical presentation, diagnostic assessment, and treatment in the present case.

Time point	Event
Day 1	Onset of right-sided nasal obstruction associated with hyposmia.
Day 13	First medical consultation. Symptoms initially attributed to allergic rhinosinusitis and managed with standard medical therapy.
Week 5	Persistent and progressively worsening nasal symptoms despite appropriate medical treatment.
Week 7	Abrupt development of rapidly progressive right-sided proptosis, prompting urgent referral to our tertiary care center.
Week 8	Comprehensive otolaryngologic and ophthalmologic assessment performed.
Week 8	Contrast-enhanced CT and MRI demonstrate an infiltrative maxillo-ethmoido-orbito-nasal mass with erosion of the ethmoidal roof and orbital invasion.
Week 9	Endoscopic-guided nasal biopsy obtained. Histopathology and immunohistochemistry confirm alveolar rhabdomyosarcoma.
Week 10	Multidisciplinary tumor board review: diagnosis classified as T2BN1M0, IRS Group III.
Week 11	Initiation of concomitant radio-chemotherapy.
1 year follow-up	Marked regression of the tumor mass on imaging with significant improvement in visual acuity and no evidence of disease progression.

### Timeline of events

On examination, the patient was alert and afebrile (37.3°C), with stable vital signs. Nasal endoscopy revealed a friable whitish polypoid mass arising from the right middle meatus, associated with diffuse mucosal inflammation and inferior turbinate hypertrophy. The nasopharynx appeared unremarkable. Cervical examination identified ipsilateral mobile laterocervical lymphadenopathy.

**Fig. 1:**
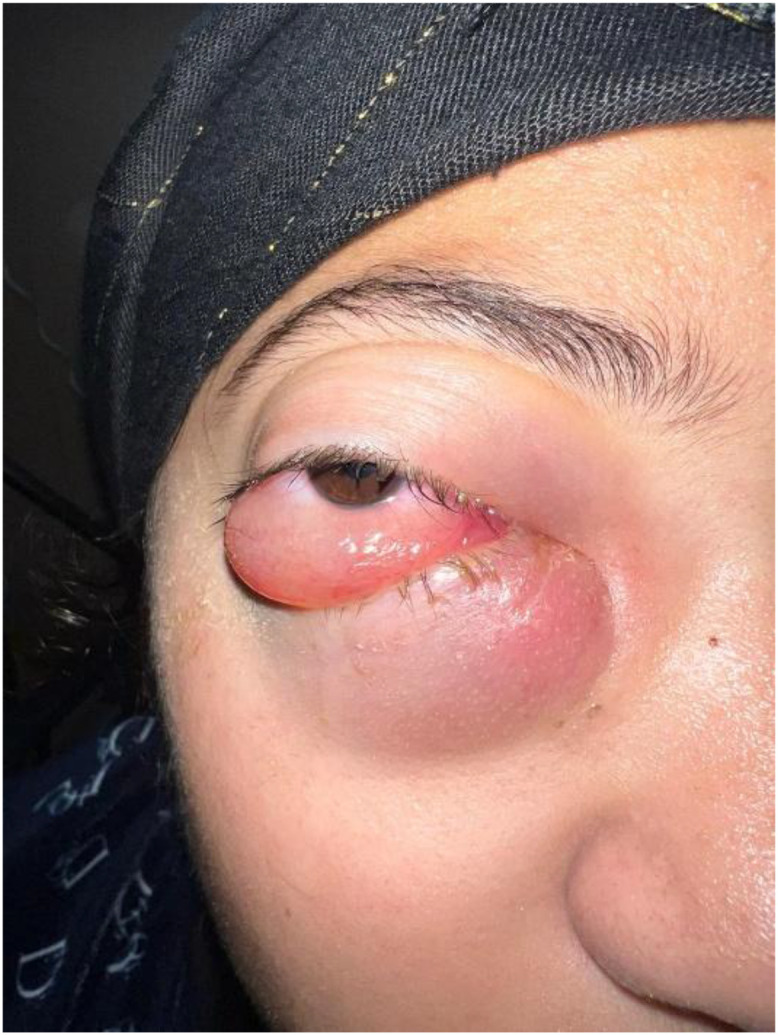
Clinical photograph showing right-sided proptosis with induration and swelling of the lower eyelid.

Ophthalmologic evaluation demonstrated grade III right proptosis with upper eyelid ptosis and lower eyelid induration (Fig. 1). Visual acuity was reduced to 5/10 in the right eye, while intraocular pressure remained normal (15 mmHg). Fundoscopic findings were within normal limits. The left eye was unremarkable.

Routine laboratory workup, including complete blood count, serum electrolytes, and bone marrow aspiration, was within normal limits.

**Fig. 2:**
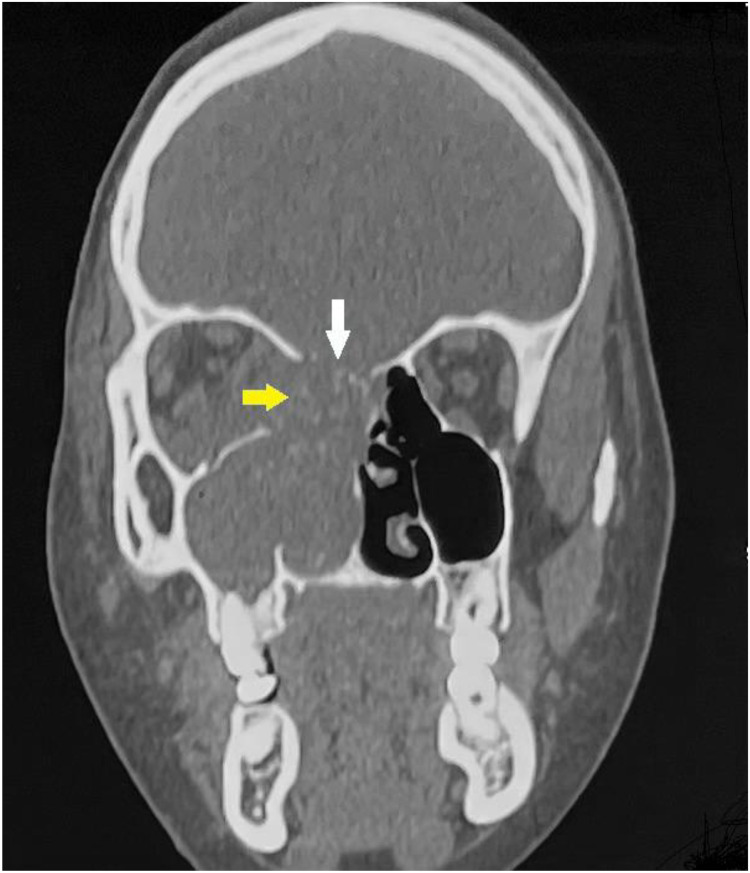
Coronal bone-window contrast-enhanced head and neck CT demonstrates a right ethmoido-maxillo-nasal mass exhibiting internal calcifications and completely occupying the ipsilateral nasal cavity. The lesion demonstrates focal osseous destruction of the lamina papyracea (yellow arrow) and erosion of the right ethmoidal roof (white arrow).

Contrast-enhanced head and neck CT demonstrated a right ethmoido-nasal mass with internal calcifications, associated with focal osseous destruction of the lamina papyracea and erosion of the ethmoidal roof, extending into the orbit (Fig. 2).

**Fig. 3:**
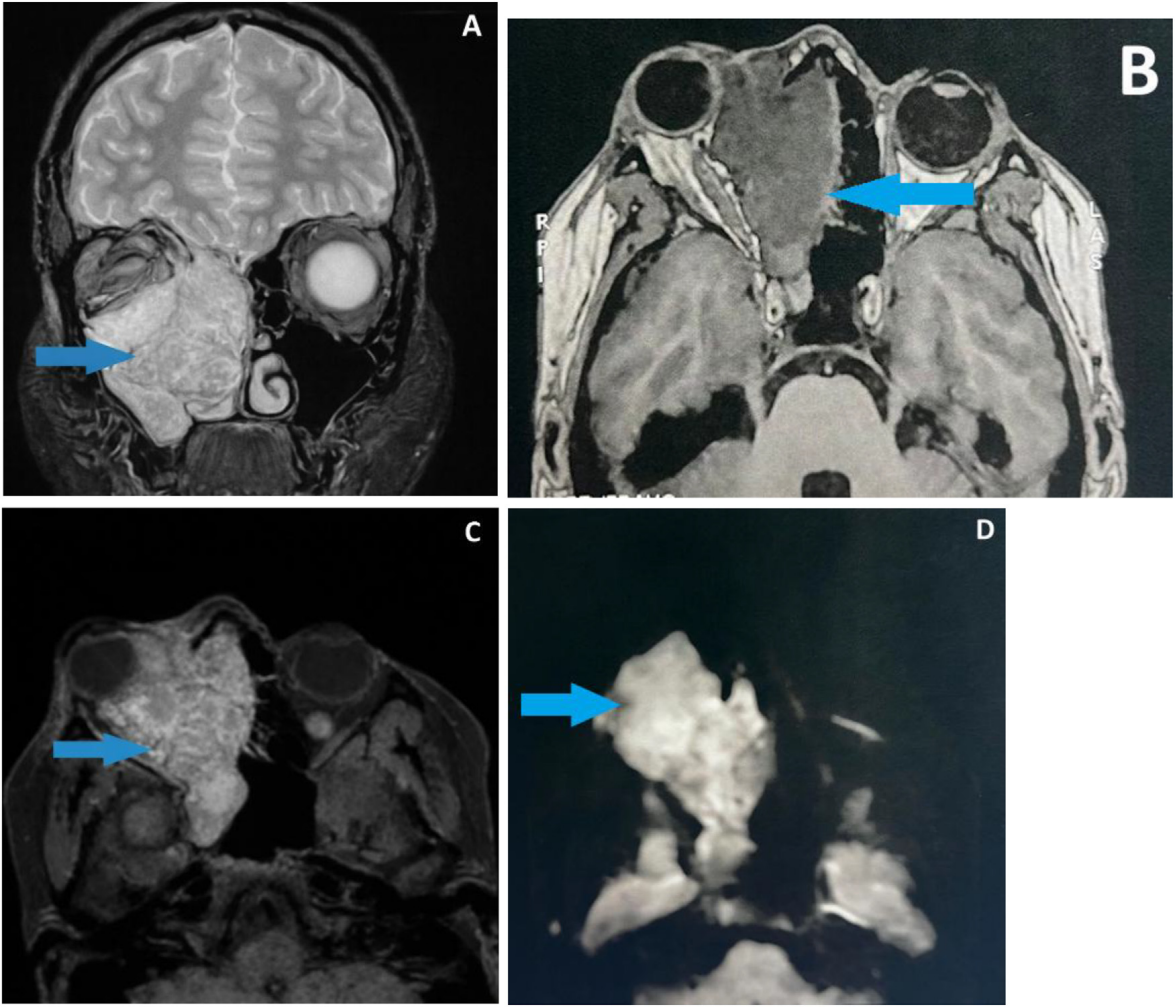
Head and neck MRI demonstrates a right maxillo-orbito-ethmoido-nasal mass (blue arrow) measuring 68 × 60 × 48 mm. The lesion appears isointense on both T2 (A) and T1-weighted (B) sequences, exhibits heterogeneous enhancement following gadolinium administration (C), and shows restricted diffusion with high signal intensity on DWI (D). The mass exerts a marked mass effect on the deformed right globe, resulting in grade III proptosis, and extends superiorly into the right basifrontal region.

Head and neck MRI revealed a large infiltrative right maxillo-orbito-ethmoido-nasal mass with ill-defined margins. The lesion showed intermediate signal intensity on both T1- and T2-weighted sequences, restricted diffusion, and heterogeneous enhancement following gadolinium administration, without cystic components. Marked mass effect on the right globe resulted in grade III proptosis, and superior extension through the cribriform plate into the basifrontal region was observed (Fig. 3). As part of the locoregional and distant staging assessment, thoraco-abdominopelvic CT performed using parenchymal, bone, and lung windows demonstrated ipsilateral laterocervical lymphadenopathy, with the largest lymph node measuring 25 mm in its greatest diameter, and no evidence of distant metastatic disease.

**Fig. 4:**
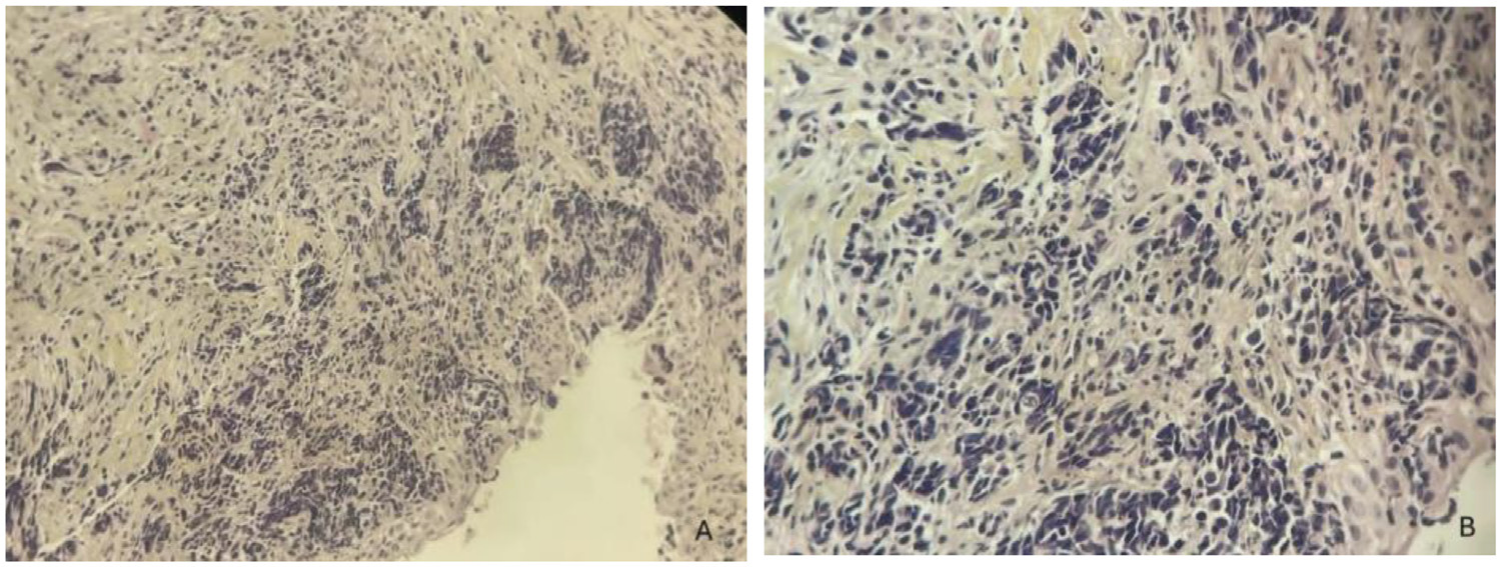
Hematoxylin and eosin stained sections at × 100 (A) and × 200 (B) demonstrating a small round hyperchromatic tumor cell arranged in fibrovascular septated nests with focal central discohesion.

**Fig. 5:**
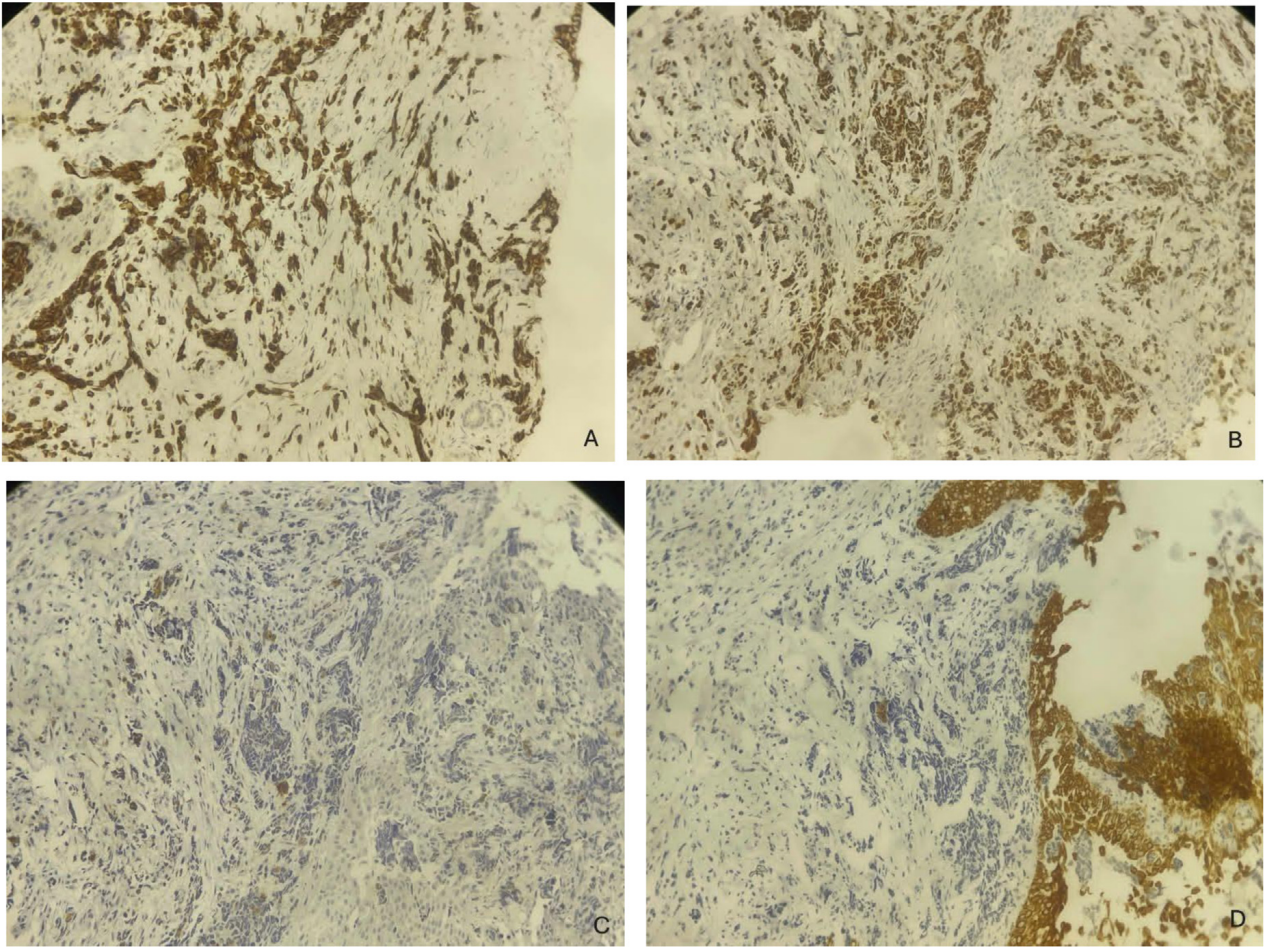
Immunohistochemical panel demonstrating robust cytoplasmic Desmin expression (A) and diffuse nuclear Myogenin positivity (B), contrasted with negative staining for Synaptophysin (C) and Pancytokeratin (D).

An endoscopic-guided nasal biopsy was performed under local anesthesia using a 30° rigid endoscope. Histopathological examination demonstrated a malignant small round cell tumor with high nuclear-to-cytoplasmic ratio and fibrovascular septation (Fig. 4). Immunohistochemical analysis demonstrated strong cytoplasmic positivity for Desmin and diffuse nuclear expression of Myogenin, while Synaptophysin and Pancytokeratin were negative, thereby confirming the diagnosis of alveolar rhabdomyosarcoma (Fig. 5).

Following MDT board review involving radiologists, pathologists, otorhinolaryngologists, ophthalmologists, and medical and radiation oncologists, the diagnosis of alveolar rhabdomyosarcoma originating from the orbit and extending into the nasosinusal cavity was subsequently confirmed. The tumor was staged as T2BN1M0, corresponding to Group III according to the Intergroup Rhabdomyosarcoma Study classification.

The patient underwent multimodal treatment consisting of vincristine, actinomycin D, and cyclophosphamide (VAC) chemotherapy combined with conformal radiotherapy (45 Gy), in accordance with the EpSSG RMS 2005 protocol. The treatment was well tolerated, with no significant adverse effects reported throughout the therapeutic course. This integrated strategy aimed to achieve maximal local tumor control while minimizing functional sequelae.

**Fig. 6:**
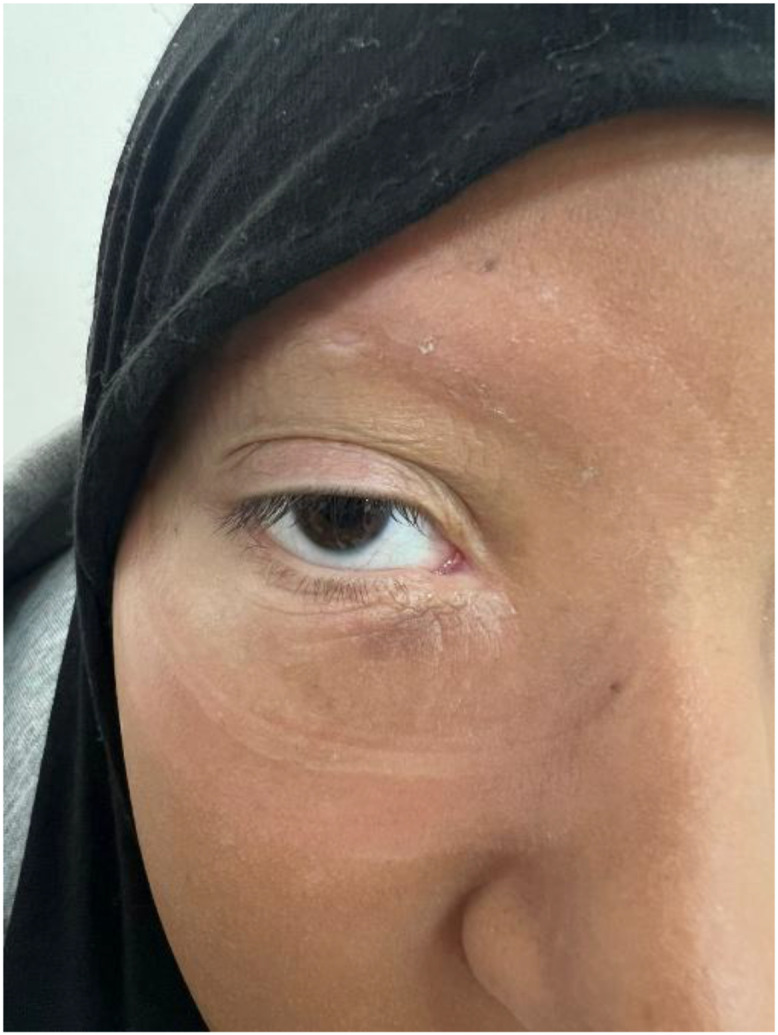
One-year post-treatment clinical photograph showing complete regression of the orbital mass, with full resolution of proptosis and restoration of normal globe position.

The patient was monitored every 3 months through systematic otolaryngologic and ophthalmologic examinations, with a cervico-thoraco-abdomino-pelvic CT scan performed every 6 months. After 1 year of follow-up, she exhibited marked clinical improvement, including complete resolution of proptosis and recovery of visual acuity to 8/10 (Fig. 6). She also reported excellent functional and aesthetic satisfaction, and no late treatment-related complications were detected during surveillance.

## Discussion

RMS is a malignant mesenchymal tumor with skeletal muscle differentiation. While it predominantly affects the pediatric population, its occurrence in adults is rare and often associated with delayed diagnosis, particularly when arising in the sinonasal or orbital regions [6]. Adult RMS generally exhibits a more aggressive behavior and carries a poorer prognosis compared with pediatric forms, partly due to intrinsic tumor resistance to therapy [7].

In the head and neck region, orbital and parameningeal localizations are particularly challenging because of their nonspecific initial presentation and complex anatomy, frequently resulting in diagnostic pitfalls [8]. The extremities are the predominant site in adults, with the trunk, genitourinary tract, and head and neck region affected less frequently [7].

In adults, RMS is exceptionally rare, particularly in the head and neck region. Among the histopathological variants, the alveolar variant is more frequently encountered in adolescents and young adults and is associated with poorer clinical outcomes [9]. This subtype is commonly characterized by the PAX3–FOXO1 fusion gene resulting from the t(2;13) (q35;q14) translocation, detected in approximately 60% of cases, which is known to drive tumor aggressiveness observed in adult alveolar RMS [4].

Our case illustrates the diagnostic challenges of orbital RMS in adults, particularly when the initial presentation mimics benign sinonasal disease. The patient presented with nonspecific rhinologic symptoms, including nasal obstruction and hyposmia, preceding the rapid onset of proptosis. Such misleading presentations are rare and can result in diagnostic delays, highlighting the importance of maintaining a high index of suspicion in adults with progressive unilateral nasal and orbital symptoms.

Similar misleading clinical presentations have been described in the literature, particularly when arises in or extends into sinonasal regions. Dilger et al. [10] reported a case of widely metastatic sinonasal alveolar RMS in an adult woman who was initially treated for presumed acute sinusitis due to months of unilateral sinus symptoms, resulting in delayed recognition of the underlying malignancy until advanced disease was evident on imaging and pathology.

Similarly, adult embryonal RMS involving the nasal cavity and paranasal sinuses has been misdiagnosed as orbital cellulitis due to overlapping clinical features such as orbital swelling and purulent rhinorrhea unresponsive to antibiotic therapy [11].

Adult orbital and sinonasal masses are diagnostically challenging due to overlapping imaging features. In our patient, initial nonspecific rhinologic symptoms delayed recognition, illustrating a misleading clinical presentation. CT Imaging features in our patient exhibited several hallmark radiologic signs that strongly suggest malignant behavior. Across published series, malignant sinonasal and orbital tumors typically demonstrate ill-defined margins, infiltrative growth, bone destruction, and extension across anatomical compartments, all of which were present in this case [12,13]. These features helped differentiate RMS from more benign or inflammatory conditions.

Esthesioneuroblastoma was initially considered due to the tumor’s ethmoidal origin and skull-base involvement. Typically, this tumor shows a superior nasal cavity origin, “dumbbell” morphology across the cribriform plate, and homogeneous enhancement on MRI [14]. This hypothesis was further supported by the patient’s significant occupational exposure to wood dust, a well-recognized risk factor for sinonasal malignancies. In contrast, MRI in our case revealed irregular thickening of the inferior rectus muscle, with heterogeneous enhancement, features more characteristic of orbital RMS. Definitive exclusion relied on histopathology and IHC, which demonstrated diffuse cytoplasmic Desmin positivity and strong nuclear Myogenin expression, with absence of neuroendocrine markers (synaptophysin negative), thereby ruling out esthesioneuroblastoma.

Sinonasal lymphoma and other small round blue-cell tumors were also considered. In our patient, the presence of cervical lymphadenopathy raised the possibility of lymphoma. Radiologically, lymphoma typically appears as a homogeneously enhancing soft-tissue mass with relative preservation of adjacent bony structures, reflecting its infiltrative growth pattern without early cortical destruction [15]. Whereas Ewing sarcoma or metastatic small-cell carcinoma may demonstrate bone destruction but can be distinguished by their characteristic immunoprofiles [16]. In our case, the absence of CD99 and CD45 expression excluded Ewing sarcoma and Lymphoma, while the lack of systemic disease argued against metastatic small cell carcinoma.

Sinonasal squamous cell carcinoma is also an important differential diagnosis in the adult population. However, it was excluded in our case by the absence of pan-cytokeratin expression. Finally, complicated sinusitis or orbital cellulitis can mimic early RMS clinically, but imaging typically shows edema without a discrete mass or bone destruction, and these conditions respond to antibiotics. Our patient’s persistent mass, aggressive imaging features, and histologic confirmation underscored the importance of early cross-sectional imaging and a multidisciplinary approach for timely diagnosis and management in adult orbital RMS.

The overlapping clinical and radiologic features observed in our patient underscore the significant diagnostic challenges posed by orbital masses extending from the sinonasal region in adults. Such presentations can mimic a broad spectrum of inflammatory, infectious, and malignant conditions, thereby increasing the risk of misdiagnosis or delayed recognition.

Management of orbital RMS in adults remains challenging due to its rarity, locally aggressive behavior, and absence of standardized treatment protocols. Most available evidence is derived from pediatric series or small adult case reports. Current literature consistently supports a multimodal therapeutic strategy, integrating systemic chemotherapy with locoregional radiotherapy and, when feasible, surgical resection, as the most effective approach for achieving disease control in patients with nodal involvement. Gerber et al., in a retrospective study of 148 patients, demonstrated that adult patients treated according to multimodality protocols had significantly improved survival compared with those managed with single-modality or non-protocol-based treatments [17]. Similarly, studies from cooperative groups have reported superior locoregional control when radiotherapy is systematically administered to both the primary tumor site and involved lymph node basins [6].

In orbital and parameningeal rhabdomyosarcoma, surgical resection is often limited by anatomical constraints and the risk of major functional sequelae. Consequently, treatment relies predominantly on chemotherapy combined with conformal radiotherapy [18].

Several chemotherapy protocols have been used in adult RMS, including IVA (ifosfamide, vincristine, actinomycin D), VDC/IE, and doxorubicin- or ifosfamide-based combinations. VAC based regimens remain the backbone of systemic treatment, while radiotherapy doses of approximately 45 to 50 Gy have been shown to provide effective local and nodal control with acceptable toxicity profiles [19]. Importantly, several series suggest that regional nodal irradiation improves disease control in patients with lymph node metastases, even in the absence of surgical lymphadenectomy [20].

Despite these advances, the prognosis of adult rhabdomyosarcoma with nodal involvement remains less favorable than in pediatric populations, with reported 5-year overall survival rates ranging between 30% and 40%, depending on histologic subtype, tumor size, and response to therapy [21].

In this context, our case is noteworthy. Despite the presence of recognized adverse prognostic factors, namely adult age, alveolar histology, large tumor size, and ipsilateral cervical lymph node involvement, the patient achieved an excellent clinical and radiologic response following VAC chemotherapy and conformal radiotherapy. Complete regression of both the primary tumor and nodal disease was observed, with sustained functional recovery and no evidence of recurrence at 1-year follow-up.

This report is limited by the relatively short duration of follow-up. Although the patient achieved an excellent clinical and radiologic response at 1 year, longer surveillance is necessary to assess long-term local control, late treatment-related toxicity, and disease-free survival.

This case emphasizes the pivotal role of imaging in raising early suspicion for orbital RMS in adults, particularly when presenting with atypical rhinologic symptoms. Recognition of aggressive radiologic features, careful consideration of differential diagnoses, and close radiologic-pathologic correlation are essential to avoid diagnostic delay. Despite unfavorable prognostic factors, timely multimodal treatment can result in excellent clinical and radiologic outcomes.

## Conclusion

Orbital rhabdomyosarcoma in adults is rare and may present with misleading sinonasal symptoms, leading to delayed diagnosis. This case highlights the crucial role of cross-sectional imaging, particularly MRI, in identifying aggressive features that should prompt early biopsy. Definitive diagnosis relies on histopathology and immunohistochemistry, and timely multimodal management can achieve excellent clinical and functional outcomes, even in high-risk adult patients.

## Patient consent

The patient gave their informed consent to the publication of this case report.
